# Draft Genome Sequence of *Romboutsia weinsteinii* sp. nov. Strain CCRI-19649^T^ Isolated from Surface Water

**DOI:** 10.1128/genomeA.00901-17

**Published:** 2017-10-05

**Authors:** Andrée F. Maheux, Dominique K. Boudreau, Ève Bérubé, Maurice Boissinot, Philippe Cantin, Frédéric Raymond, Jacques Corbeil, Rabeea F. Omar, Michel G. Bergeron

**Affiliations:** a Centre de Recherche en Infectiologie de l’Université Laval, Axe Maladies Infectieuses et Immunitaires, Centre de echerche du CHU de Québec–Université Laval, Québec City, Québec, Canada; b Direction de l’eau potable et de l’eau souterraine, Ministère du Développement durable, de l’environnement et de la Lutte contre les Changements climatiques, Québec City, Québec, Canada; c Centre de recherche en données massives, Université Laval, Québec City, Québec, Canada; d Département de Microbiologie, infectiologie et d'immunologie, Faculté de Médecine, Université Laval, Québec City, Québec, Canada; e Département de Médecine moléculaire, Université Laval, Québec City, Québec, Canada

## Abstract

*Romboutsia weinsteinii* sp. nov. CCRI-19649^T^ belongs to the genus *Romboutsia*. The strain was isolated from a water sample harvested in Québec City, Québec, Canada. The genome assembly comprised 4,134,593 bp with a 29.3% GC content. This is the first documentation that reports the genome sequence of *R. weinsteinii*.

## GENOME ANNOUNCEMENT

During a study comparing the ability of the membrane *Clostridium perfringens* (mCP) agar culture-based method and CRENAME (*c*oncentration *r*ecovery *e*xtraction of *n*ucleic *a*cids and *m*olecular *e*nrichment) alpha-toxin-specific real-time PCR assay to detect *Clostridium perfringens* spores in drinking water, 147 putative *C. perfringens* colonies were harvested from surface and river water collected in Québec City, Québec, Canada (1). These colonies were then subjected to PCR and DNA sequencing of the 16S rRNA gene for species identification. Comparative 16S rRNA gene sequence analysis showed that the closest cultured relative of strain CCRI-19649^T^ was *Romboutsia ilealis* CRIB^T^ (98.0%; GenBank accession number LN555523, position 2,388,619 to 2,390,111) (2). The genome of *R. ilealis* CRIB^T^ was also compared to strain CCRI-19649^T^ for a genomic relatedness assessment (accession number LN555523). The genomic average nucleotide identity (ANIb) (3) obtained was 75.3%. Since ANIb values around 95% corresponded to the 70% DNA-DNA hybridization cutoff value for species discrimination, strain CCRI-19649^T^ represents a novel species of the genus *Romboutsia* (4).

*R. weinsteinii* is a rod-shaped, strictly anaerobic, and spore-forming bacterium. It grows on blood agar in 24 h at 35°C, incubated in an anaerobic atmosphere as previously described (5), forming 2-mm convex gray colonies. Genomic DNA was isolated by using the BioSprint 15 DNA blood kit (Qiagen) automated with a KingFisher mL instrument (Thermo Fisher Scientific). Whole-genome sequencing of strain CCRI-19649^T^ was performed on an Illumina HiSeq 2500 platform using SBS version 4 to sequence a 126-bp paired-end library (Nextera XT, Illumina). A total of 17,688,325 reads were assembled *de novo* in 151 contigs using Ray version 2.3.0 software (6). The total genome length is 4,134,593 bp (*N*_50_ value of 45,211 bp) with an average G+C content of 29.3% (7). The draft genome sequence was annotated using the NCBI GenBank version 4.2 annotation pipeline and the RAST version 2.0 annotation server (8). A total of 4,078 features were identified, including 27 rRNAs (complete or partial) and 62 tRNAs. Of the 3,950 putative protein-coding sequences, 1,524 were assigned as hypothetical proteins.

*R. weinsteinii *(weins.tein’i.i. N.L. masc. gen. n. weinsteinii) was named after Louis Weinstein (1908−2000), a physician and founder of the modern medical specialty of infectious diseases and a leading researcher on antibiotherapy at Boston medical schools, imparting his clinical wisdom to generations of students and teaching them to diagnose the etiologic agent of an infectious process, not a disease (9).

### Accession number(s).

This whole-genome shotgun project of *R. weinsteinii* CCRI-19649^T^ has been deposited at DDBJ/ENA/GenBank under the accession number NOJY00000000. The version described in this paper is the first version, NOJY01000000.
